# Assessing the impact of vector control interventions by measuring their effectiveness - what has been done in Madagascar

**DOI:** 10.1186/1475-2875-13-S1-O5

**Published:** 2014-09-22

**Authors:** Thomas Kesteman, Milijaona Randrianarivelojosia, Patrice Piola, Christophe Rogier

**Affiliations:** 1 Institut Pasteur de Madagascar, Antananarivo, Madagascar

## 

The impact of disease control can be either evaluated by a classical ecological study, or by multiplication of the effectiveness of control intervention and the disease burden. The latter approach has the advantage of circumventing ecological biases. To evaulate the effectiveness of the malaria control program implemented in Madagascar, we conducted a nationwide survey in 2012-2013 in 62 study sites. This survey included (1) a cross-sectional study to measure the effectiveness of each control intervention on reducing the transmission, and (2) a case-control study to measure the effectiveness on reducing the morbidity. We present here the results related to vector control interventions, i.e. Long Lasting Insecticidal Nets (LLIN) distribution and Indoor Residual Spraying (IRS) campaigns.

The cross-sectional survey included 15,734 individuals of all age groups among which 3.7% had a positive Rapid Diagnostic Test (RDT). LLIN daily use was 52.3% in areas covered by universal distribution and IRS coverage was 64.8% in targeted areas. 818 uncomplicated clinical malaria cases were compared to 7,767 controls living in the same villages. Multilevel analysis of factors associated with a positive RDT or with the occurrence of an episode of non-complicated malaria revealed that LLIN daily use had a 45% protective effectiveness (PE) against infection (aOR 0.55 [95% CI 0.42, 0.72]) and a 48% PE against morbidity (aOR 0.52 [0.28, 0.96]). The PE of IRS was evaluated to be 23% against infection (aOR 0.77 [0.53, 1.13]) and 49% against morbidity (aOR 0.51 [0.39, 0.66]). In areas where both activities are implemented, coverage of LLIN was 21.3 percentage points lower than in areas where LLIN only were deployed. Combining IRS with LLIN provided almost no gain in preventing infection, but the PE of LLIN use against morbidity increased from 51% (aOR 0.49 [0.20, 1.20]) to 66% (aOR 0.34 [0.16, 0.74]) when IRS was added, although non significantly.

Our results indicate that, taken separately, LLIN and IRS perform satisfactorily but that their concurrent use might have a limited benefit as compared with efforts to improve the coverage of a single intervention. Given that in Madagascar, approx. 220,000 clinical malaria cases and 770,000 malaria infections occur each year, we calculated that vector control interventions prevented approx. 96,000 malaria cases and 197,000 malaria infections annually. Distributions of LLIN were implicated in the major part of cases (73.7%) and infections (80.5%) prevented, while IRS campaigns were implicated in 37.3% of cases and 31.2% of infections prevented.

